# Automated Implantable Cardioverter Defibrillator/Pacemaker Lead Fracture

**DOI:** 10.7759/cureus.11562

**Published:** 2020-11-19

**Authors:** Jose Rubero, Sidhartha R Ramlatchan, Matthew Solomon, Andrew George, Latha Ganti

**Affiliations:** 1 Emergency Medicine, University of Central Florida College of Medicine, Orlando, USA; 2 Emergency Medicine, Envision Physician Services, Plantation, USA; 3 Emergency Medicine, Osceola Regional Medical Center, Kissimmee, USA; 4 Emergency Medicine, HCA Healthcare Graduate Medical Education Consortium Emergency Medicine Residency Program of Greater Orlando, Orlando, USA; 5 Emergency Medicine, Drexel University, Philadelphia, USA; 6 Emergency, Brown University, Providence, USA; 7 Emergency Medicine, Brown University, Providence, USA

**Keywords:** pacemaker lead fracture, electrophysiology, automatic implantable cardioverter defibrillator

## Abstract

Implanted artificial pacemakers are groundbreaking pieces of technology that have a vast array of medical benefits. However, as with other electronic devices, these implanted cardiac devices are not immune to failure. One of the most common failures are lead fractures, which can lead to conduction issues that result in inappropriate or insufficient electrical stimulation to the myocardium or other myocytes. The authors present a classic example of this type of artificial pacemaker failure, with the hospital course of a female patient presenting with erratic muscle contractions due to improper electrical impulse generation and conduction.

## Introduction

Under normal circumstances, pacemaker cells in the heart can regulate heart rate and rhythm. These cardiomyocytes are concentrated in three main locations - the sinoatrial (SA) node, the atrioventricular (AV) node, and the Bundle of His and Purkinje fibers together. Each concentration exhibits a lower degree of automaticity, such that spontaneous depolarization takes longer in the Bundle of His/Purkinje fibers than the AV node, which in turn takes longer than the SA node. Thus, under normal circumstances, the SA node regulates cardiac depolarization and contraction, with other pacemaker cells serving as backups. Despite this redundancy, however, the lower degrees of automaticity in ectopic pacemakers make SA node failure a serious issue.

Cardiac implantable electronic devices (CIEDs) are used internally to correct or monitor arrhythmia. These devices, including pacemakers, implantable cardioverter defibrillators (ICDs), and implantable cardiac loop recorders [1,2] are commonly used in patients at risk of heart failure and sudden cardiac arrest.

While artificial cardiac pacemakers issue electric impulses to treat bradycardia, ICDs deliver an electric shock to depolarize heart muscle, decreasing one’s heart rate [3]. Modern ICDs are typically dual-purposed also completing the functions of a pacemaker. These devices are surgically implanted and require a battery change about every seven years. Alternatively, implantable cardiac loop recorders are used solely to record subtle cardiac abnormalities often overlooked by a standard electrocardiogram (ECG).

In cases where the automaticity of the heart is no longer sufficient, an artificial cardiac pacemaker is often implanted. Though there are multiple variations of such a device, the overall intent is to regulate the electrical conduction system of the heart when the natural pacemaker is either completely nonfunctional or otherwise insufficient (for instance if the natural pacemaker would result in chronic bradycardia).

Though the specific configuration of a pacemaker can be programmed to be patient specific, the overall concept involves sensory and stimulus functions. Pacemakers can detect and monitor electrical activity to ensure proper rhythm, rate, synchronicity, and so on. Should one of these parameters fail, the pacemaker generator can deliver, through the leads, an electrical impulse to specific regions of the heart as necessary to ensure proper function. A smaller, leadless pacemaker is available for a limited number of conditions as well, where a smaller generator is implanted directly into the cardiac wall.

One of the most common complications of having a pacemaker is lead fracture. This occurs in roughly 1-4% of patients with pacemakers [4]. Often as a result of weightlifting or chest trauma, lead fractures are characterized by the damaging of one or more pacemaker leads. This is one of the reasons why patients with pacemakers are told to avoid heavy lifting and stress in the chest area. Lead fractures often occur near the subclavian venous entry site as a result of compression between the clavicle and ribs. Patients experiencing loss of consciousness, chest pain or discomfort, dizziness and palpitations should immediately consult their physician in case of lead fracture. The diagnosis is then made by careful review of chest radiography and ECG results [5]. Lead fracture is a particularly urgent issue for patients who heavily depend on their pacemakers for normal cardiac function. 

We present such a case, where an 80-year-old female with a history of cardiac issues presented with regular, involuntary muscle contractions after a high-velocity theme park ride. In this case, the fracture occurred during amusement park rides and went undiagnosed until the patient presented for a routine pacemaker evaluation.

## Case presentation

An 80 year-old female presented to the emergency department with a chief complaint of repeated twitching in her upper chest and arm. The patient and her family reported that this had been going on for the past three days, however there was no accompanying chest pain. The patient reported a history of asthma, hypertension, congestive heart failure, and diabetes mellitus. The patient also had an automatic implantable defibrillator (AICD).

On presentation, these twitches were observed to occur rhythmically and regularly, in approximately 3-5 second intervals, in the bicep, shoulder, and upper chest on her left side. The patient’s skin and soft tissue around the AICD were normal, with no signs of erythema, tenderness, or swelling, and no palpable hematoma.

**Figure 1:**
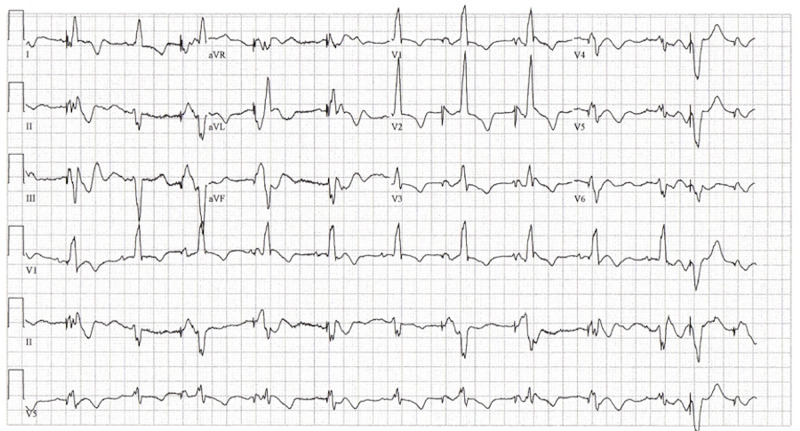
Patient's electrocardiogram

Her initial vital signs were: pulse 74 beats per minute; blood pressure 120/54 mmHg; respiratory rate 18 breaths per minute; temperature 36.3^0^C; O_2 _Sat 97%. Her physical examination was unremarkable except for an AICD in the upper left chest wall. Initial blood work showed a white blood cell count of 7.42 x10^3^/m^3^, hemoglobin of 12.1 g/dL, sodium 136 mmol/L; potassium 5.4 mmol/L; Cl 103 mmol/L; HCO3 31 mmol/L; BUN 37 g/dL; creatinine 1.86 g/dL; glucose 204 mg/dL; Troponin I <0.02 ng/ml. An electrocardiogram was obtained showing an intermittent atrial pacing at 100 beats per minute, left axis deviation, artifacts, premature ventricular contractions, right bundle branch block, non-specific diffused ST-T changes; and no ST elevations (Figure 1).

**Figure 2:**
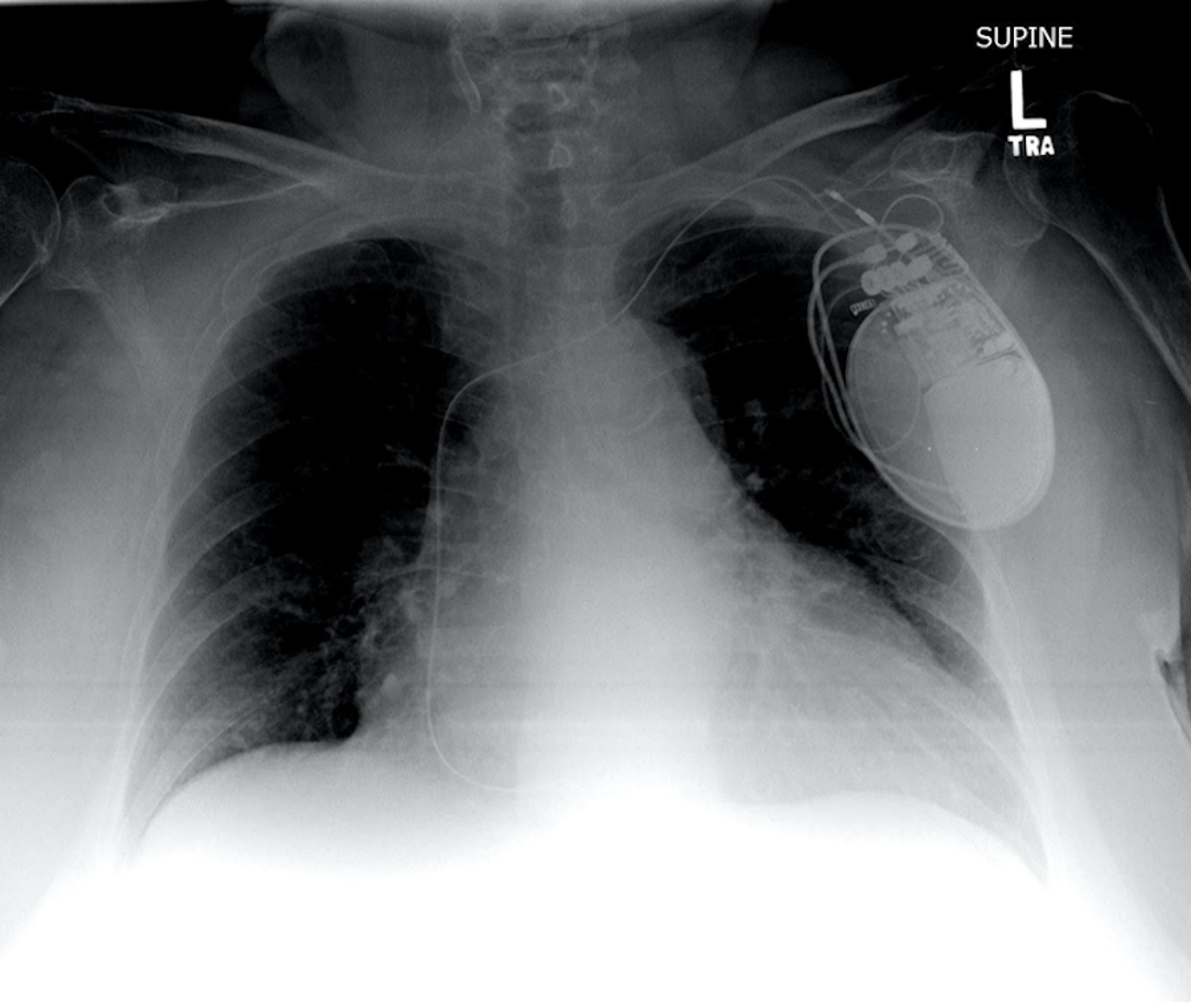
Supine portable radiograph reveals a fractured and coiled lead on the upper portion of the device

Upon consultation with a representative of the AICD manufacturer to interrogate the device, the contractions were noted to be occurring in conjunction with its firing. Supine portable chest x-ray revealed a fractured and coiled lead on the upper portion of the device (Figure 2).

The AICD device representative reprogrammed the device, ceasing its firing, and accordingly the patient’s muscle twitching ceased. The patient was admitted and care transferred to an electrophysiological cardiologist to remove the fractured wire and replace the AICD.

## Discussion

**Figure 3:**
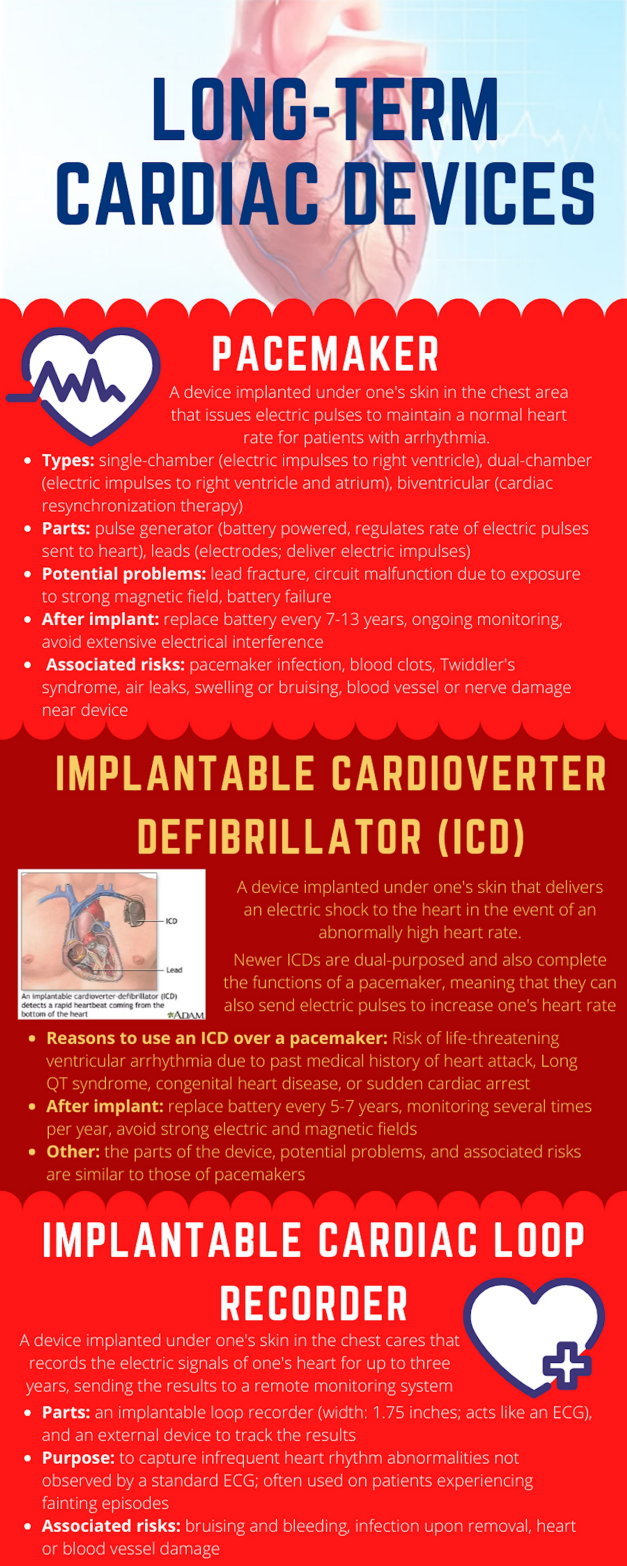
Long term cardiac devices

Implantable cardioverter defibrillators (ICD) are one type of long term cardiac device (figure 3).

Conductor fractures are common forms of lead failure in which an ICD’s pace sense components are affected. An ICD must be interrogated and have its functionalities and measurements reviewed in order for the correct type of lead failure to be determined and for the correct procedures to be carried out on the device. Certain measured abnormalities can exist in a lead that indicate a conductor fracture [6]. High levels of, and abnormally high increases in impedance can be detected by an ICD and suggest that a malfunction (such as a conductor fracture) may be preventing a full current in the lead [7]. Our patient was found to have a broken atrial lead most likely secondary to pacemaker-twiddler syndrome. This syndrome is an uncommon cause of lead fracture that occurs due to unintentional or deliberate manipulation of the pacemaker pulse generator within its skin pocket by the patient [8]. 

Inappropriate defibrillator shocks made by the ICD can serve as another indication of a conductor fracture. Electrical artifacts generated by the malfunctioning leads (and other noncardiac electrical signals) can commonly be misinterpreted as ventricular fibrillation by the ICD, triggering defibrillation impulses. Alternatively, oversensing can occur when misinterpreted noncardiac signals cause the ICD system to inhibit pacing, forcing the patient’s heart rate to be lower than the preset rate [9].

Before an ICD is interrogated and repaired, the device must first enter an asynchronous mode. To do this, a donut magnet is applied to the ICD, allowing for cardiac signal detection to be disabled while procedures are being done on the machine [10]. Lead fractures require an additional lead to be installed on the ICD - the fractured lead can either be removed or left inside the patient. This decision depends on specific factors related to the patient and their ICD, such as the age of the patient, the age and functionality of the fractured lead, the number of leads in the ICD [6]. In many cases, the fractured lead is capped and left inside of the patient in an effort to prevent potential lead extraction complications such as cardiac perforation and arrhythmias [5]. However, the complications of leaving a fractured lead inside of a patient (such as vein blockage and interference with working leads) must also be taken into account.

## Conclusions

ICD interrogation is imperative for a correct diagnosis of lead fractures and other types of lead failures. In general, AICDs should be interrogated every three months. When a fractured lead is being repaired, it is important to use known information about both the patient and the lead itself to decide if the potential complications of the surgery are worth the risks associated with removing a fractured lead from a patient as opposed to leaving it in their body. These complications drive the ongoing research towards producing leadless pacemakers.
